# Long-term follow-up of complete remission in a patient with advanced hepatocellular carcinoma treated with sorafenib: a case report

**DOI:** 10.3389/fonc.2023.1260989

**Published:** 2023-10-10

**Authors:** Gordan Adžić, Juraj Prejac, Stjepko Pleština

**Affiliations:** ^1^ Department of Oncology, University Hospital Centre Zagreb, Zagreb, Croatia; ^2^ Department of Pathophysiology, School of Dental Medicine, University of Zagreb, Zagreb, Croatia; ^3^ Department of Pathophysiology, School of Medicine, University of Zagreb, Zagreb, Croatia

**Keywords:** hepatocellular carcinoma, sorafenib, complete response, tyrosine-kinase inhibitor, case report

## Abstract

**Introduction:**

Hepatocellular carcinoma (HCC) accounts for approximately 90% of primary liver cancer and can be caused by well-known risk factors, including infection with hepatitis B and C viruses, alcohol intake, and metabolic syndrome. The overall prognosis remains poor with a median survival of 1 year for symptomatic advanced-stage cases treated with systemic therapies.

**Case description:**

In July 2020, a 73-year-old male patient presented at our institution with mild abdominal pain and an attack of intense cold. After a radiological workup, the diagnosis of HCC located in the caudate lobe was established. The patient underwent atypical caudate lobe resection, and pathology confirmed the diagnosis of grade 3 HCC. Postoperative MRI showed a new metastasis in the 6th liver segment 1.3 cm in diameter, and a PVT progression which now affected the whole right lobe. The patient was started on sorafenib and demonstrated a complete response which still lasts for more than two years.

**Conclusion:**

We present a rare case of a patient who demonstrated a complete response to sorafenib treatment in advanced HCC with unfavorable prognostic factors.

## Introduction

Hepatocellular carcinoma (HCC) accounts for approximately 90% of primary liver cancer and can be caused by well-known risk factors which include infection with hepatitis B and C viruses, alcohol intake, and metabolic syndrome (1). According to the World Health Organization GLOBOCAN database, hepatocellular carcinoma (HCC) is the world’s fourth leading cause of cancer-related deaths (2). The overall prognosis remains poor with a median survival of 1 year for symptomatic advanced-stage cases treated with systemic therapies (3). Sorafenib is a multitargeted oral tyrosine-kinase inhibitor that inhibits Raf kinase and vascular endothelial growth factor receptors and is established as a first-line agent in the systemic treatment of advanced HCC. The multicenter European SHARP trial showed that overall survival, the primary endpoint, was significantly longer in the sorafenib-treated patients, however, objective response rates were low (2%) with no cases of complete response (CR) being established (4). Based on a more recent report, the CR rate for patients treated with sorafenib is less than 1% (5). A more recent study, IMbrave 150 which compared atezolizumab and bevacizumab with sorafenib in unresectable HCC demonstrated complete response in just 1 patient (less than 1%) in the sorafenib arm (6). Although IMbrave150 positioned atezolizumab and bevacizumab as the first-line choice in the treatment of HCC, adequate choice of the second-line treatment still remains a challenge (7).

Considering that, we report an infrequent case of a patient treated with sorafenib for advanced HCC resulting in a complete remission that has been maintained for almost two years.

## Case description

**Figure 1:**
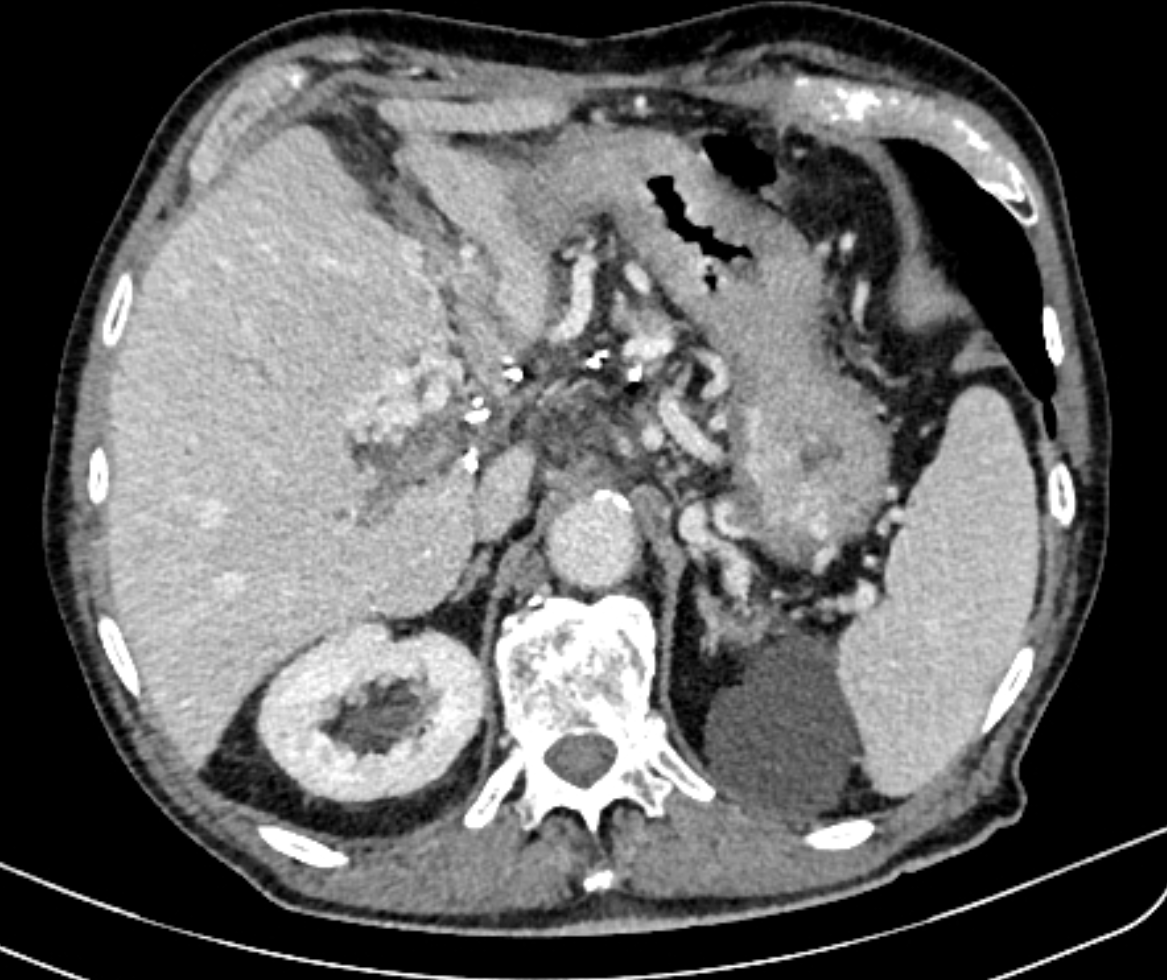
CT scan showing thrombosis of portal, splenic and superior mesenteric vein with a probable tumor thrombus.

**Figure 2:**
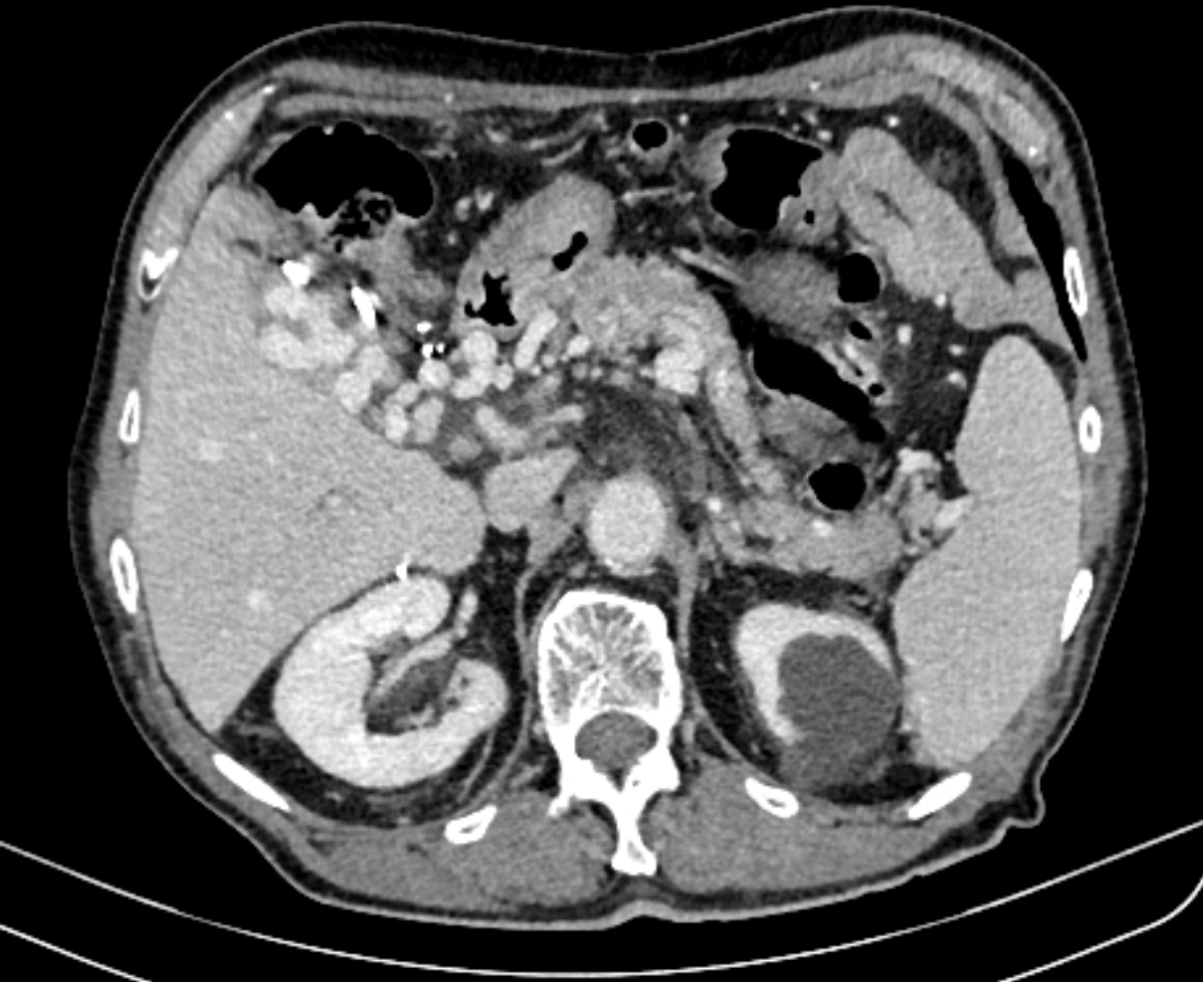
CT scan showing an expansive process of caudate lobe 5.4x3.7 cm in diameter next to portal vein thrombosis and a fatty infiltration of the rest of the liver parenchyma.

In July 2020, a 73-year-old male patient presented at our institution with mild abdominal pain and an attack of intense cold. Due to previously known urolithiasis, ultrasound, and MSCT scan were performed. These examinations revealed thrombosis of the portal, splenic, and superior mesenteric vein with a probable tumor thrombus which was inseparable from an irregular caudate lobe (**Figure 1**). Furthermore, an MRI of abdominal organs substantiated the diagnosis of HCC by showing an expansive process of caudate lobe 5.4x3.7 cm in diameter next to portal vein thrombosis (PVT) and fatty infiltration of the rest of the liver parenchyma (**Figure 2**). Besides non-alcoholic fatty liver disease (NAFLD), the patient had no other known risk factors (HBV and HCV serology were negative and the patient didn’t consume alcohol). Liver function was well retained without clinical signs of liver impairment (Child-Pugh score A6), and the patient was in good clinical condition (ECOG PS 0, BCLC C). AFP level was mildly elevated at 17.3 μg/L (nv <7) with other laboratory parameters within normal limits.

The patient underwent atypical caudate lobe resection, and pathology confirmed the diagnosis of poorly differentiated grade III HCC. R0 resection was performed, histologic stage pT4N0Mx, and the immunohistochemistry staining was positive for Hepatocyte and Glypican-3, whereas CK19 and CK7 were negative. The specimen was subjected to comprehensive genomic analysis via the FoundationOne CDx method utilizing next-generation sequencing. Findings revealed that the tumor exhibited microsatellite stability accompanied by a low tumor mutational burden of 4 mutations per megabase. Furthermore, genetic alterations in CTNNB1 and TERT genes were identified, suggesting their potential as activating mutation, however, presently there are no targeted therapeutic interventions available for these specific mutations.

The patient underwent atypical caudate lobe resection, and pathology confirmed the diagnosis of grade 3 HCC. The postoperative level of AFP declined to normal values of 4.7 μg/L. MRI showed a postoperative collection in the place of resected caudate lobe, a new metastasis in the 6th liver segment 1.3 cm in diameter, and a PVT progression which now affected the whole right lobe. From October 15, 2020, the patient started with an oral administration regimen of sorafenib, commencing at an initial dosage of 800 milligrams daily, divided into two equal doses of 400 milligrams each, administered twice daily which was recommended as the first-line treatment at the time by both the European Society for Medical Oncology (ESMO) and the National Comprehensive Cancer Network (NCCN).Due to portal thrombosis, which could not be surgically removed, the patient received daily dalteparin sodium, 12 000 IU, IM treatment, from surgery until the thrombus turned into cavernous formations. After two months, the patient was reevaluated with a new CT scan which showed partial regression of postoperative collection and PVT and complete regression of metastasis in the 6th liver segment. The patient was evaluated every two months with no signs of a viable tumor on CT scans. AFP remained lower than 5 μg/L and other laboratory parameters were normal. After the first cycle of sorafenib, the patient reported the emergence of a new maculopapular rash on both of his lower extremities accompanied by pruritus. Atypical dermatological changes were localized on both lower legs and alleviated by daily application of preparation with 10% urea. The accompanying itching was treated with loratadine PO. The observed rash was classified as CTCAE (Common Terminology Criteria for Adverse Events) grade 1. After completion of the sixth cycle, the patient presented with complaints of diarrhea which escalated in frequency and severity leading to a weight loss of 18 kilograms. Diarrhea was characterized as CTCAE grade 2 but after the introduction of loperamide and adjusting the dose of sorafenib to 400 milligrams daily, administered as 200 milligrams twice per day, the adverse event was well-managed. On a CT scan performed in May 2022, a new nodule in lungs 6 millimeters in size suspecting a new metastasis was seen, but sorafenib therapy was continued. On a CT scan performed in July 2022; the aforementioned suspect metastasis was described as a ground-glass nodule. The last MRI scan was performed in April 2023, the patient still had no signs of active disease and continued with 400 mg of sorafenib.

## Discussion

Until 2008, no effective therapy existed for patients with advanced-stage HCC. However, the introduction of sorafenib changed the landscape in the treatment of disseminated HCC. Sorafenib is a small multikinase inhibitor of the vascular endothelial growth factor receptor, the platelet-derived growth factor receptor, and Raf. In two large multicenter stage 3 trials, sorafenib showed improved progression-free and overall survival versus placebo (4, 8). Although sorafenib improves survival, one of the biggest drawbacks is the low overall objective response rate, as neither study achieved complete remission (CR) in advanced HCC (4, 8). One patient had CR in the sorafenib arm in IMbrave150, a trial comparing atezolizumab plus bevacizumab versus sorafenib (6).

After more than a decade of using sorafenib in an advanced disease setting, a complete response remains rare. According to retrospective studies, less than 1% of patients achieve a complete response, and that group of patients is quite heterogeneous in etiology and clinical manifestations of the disease (5).

Several previous studies have tried to predict which factors could lead to a response to sorafenib therapy. One of the hypothesized factors was the presence of dermatologic toxicity (5). Our patient did not have a typical hand-foot skin reaction, just an atypical rash which was alleviated with symptomatic treatment. A second described factor was diarrhea (9). Our patient had diarrhea which ultimately led to a dose reduction. According to Bruix et al., and based on *post hoc* analysis of two randomized phase 3 trials, predictive factors for response to sorafenib are HCV infection, absence of extrahepatic spread (EHS), and low neutrophil-lymphocyte ratio (NLR) (10). We found this interesting because our patient had no evidence of HCV infection, had EHS due to PVT, and had moderately high NLR (8) at the time of diagnosis. Predictive factors leading to a complete response to sorafenib are not yet well understood and warrant closer scientific scrutiny. Further studies in both HCC oncoproteomics and molecular basis are also needed to better understand treatment efficacy and resistance mechanisms (11).

The main problem that arises is the duration of sorafenib administration following CR. There is limited data regarding the long-term clinical course and management of the patients who achieved CR, especially because the recurrence rate after the discontinuation of sorafenib seems high (12). However, since sorafenib targets multiple tyrosine-kinase receptors, it can cause a wide range of side effects, especially cardiovascular toxicity which is well known in patients with renal cell cancer and lung cancer who have been receiving it for a longer period.

At the time the patient began therapy, only two systemic therapies including sorafenib (1, 8) and regorafenib (13) had been approved and reimbursed by the national health insurance in Croatia. And although we now routinely perform comprehensive genomic profiling in unresectable and metastatic solid tumors, most molecular alternations in HCC remain undruggable (14). In this particular case, FoundationOne testing revealed *CTNNB1* mutation, associated with β-catenin signaling activation (15) and no specific targeted therapy.

Finally, the IMbrave150 study demonstrated the clear superiority of atezolizumab and bevacizumab versus sorafenib, so the position of sorafenib as the sole agent for treatment for advanced HCC has changed and remains a first-line choice only for patients who have a contraindication to the combination of atezolizumab and bevacizumab (6). With that in mind, the choice of second-line treatment remains controversial since there is a lack of head-to-head trials, although a lot of new therapeutical options including TKIs and immunotherapy are being developed. Meta-analysis by Solimando et al. suggests the use of regorafenib and cabozantinib as second-line treatments in HCC, but while the emerging second-line therapies for HCC offer hope, it is crucial to validate findings through studies and address the practical challenges of cost, access to treatment and adequate sequencing of therapy (7).

In conclusion, we present a rare case of a patient who demonstrated a complete response to sorafenib treatment in advanced HCC with unfavorable prognostic factors. The limitation of our case report is inherent to a single case described, as well as the duration of the follow-up. Further studies that aim to clear mechanisms and predictive factors leading to CR are warranted to better understand long-time effectiveness, as well as to correctly position the use of sorafenib in the context of arising of more effective therapies.

## Data availability statement

The original contributions presented in the study are included in the article/supplementary material. Further inquiries can be directed to the corresponding author.

## Ethics statement

The studies involving humans were approved by University Hospital Centre Zagreb Ethics Committee, Kišpatićeva 12, Zagreb 10000, Croatia. The studies were conducted in accordance with the local legislation and institutional requirements. Written informed consent for participation was not required from the participants or the participants’ legal guardians/next of kin in accordance with the national legislation and institutional requirements. Written informed consent was obtained from the individual(s) for the publication of any potentially identifiable images or data included in this article.

## Author contributions

GA: Conceptualization, Writing – original draft. JP: Conceptualization, Investigation, Supervision, Writing – review & editing. SP: Supervision, Writing – review & editing.
